# Virus survey in populations of two subspecies of bent-winged bats (*Miniopterus orianae bassanii* and *oceanensis*) in south-eastern Australia reveals a high prevalence of diverse herpesviruses

**DOI:** 10.1371/journal.pone.0197625

**Published:** 2018-05-24

**Authors:** Peter H. Holz, Linda F. Lumsden, Julian Druce, Alistair R. Legione, Paola Vaz, Joanne M. Devlin, Jasmin Hufschmid

**Affiliations:** 1 Department of Veterinary Biosciences, Melbourne Veterinary School, The Faculty of Veterinary and Agricultural Sciences, The University of Melbourne, Werribee, Victoria Australia; 2 The Asia Pacific Centre for Animal Health, The Faculty of Veterinary and Agricultural Sciences, The University of Melbourne, Parkville, Victoria, Australia; 3 Arthur Rylah Institute for Environmental Research, Department of Environment, Land, Water and Planning, Heidelberg, Victoria, Australia; 4 Victorian Infectious Diseases Reference Laboratory, Doherty Institute, Melbourne, Australia; University of Reunion Island, RÉUNION

## Abstract

While bats are often viewed as carriers of infectious disease agents, little research has been conducted on the effects these potential pathogens may have on the bat populations themselves. The southern bent-winged bat (*Miniopterus orianae bassanii*) is a critically endangered subspecies endemic to south-eastern Australia. Population numbers of this bat have been declining for the past 50 years, but the reasons for this are unclear. As part of a larger study to determine if disease could be a contributing factor to this decline, 351 southern bent-winged bats from four locations were captured, and oral swabs were collected and tested for the presence of potentially pathogenic viruses. Results were compared with those obtained from 116 eastern bent-winged bats (*Miniopterus orianae oceanensis*) from three different locations. The eastern bent-winged bat is a related but more common and widespread subspecies whose geographical range overlaps partly with southern bent-winged bats. Herpesviruses were detected in bent-winged bats from all seven locations. At least six novel herpesviruses (five betaherpesviruses and one gammaherpesvirus) were identified. The prevalence of herpesvirus infection was higher in eastern bent-winged bats (44%, 51/116), compared to southern bent-winged bats (27%, 95/351), although this varied across the locations and sampling periods. Adenoviruses and a range of different RNA viruses (lyssaviruses, filoviruses, coronaviruses and henipaviruses) were also tested for but not detected. The detected herpesviruses did not appear to be associated with obvious ill health, and may thus not be playing a role in the population decline of the southern bent-winged bat. The detection of multiple novel herpesviruses at a high prevalence of infection is consistent with our understanding of bats as hosts to a rich diversity of viruses.

## Introduction

Historically, infectious diseases were believed to be of minimal significance in species population declines. An International Union for Conservation of Nature (IUCN) report listed 833 species extinctions over the past 500 years and attributed only 3.7% of these to infectious disease [1]. This low percentage of extinctions caused by infectious disease may be partly due to the past use of less sophisticated diagnostic techniques to detect infectious agents, as it is now becoming apparent that the effects of urbanization, human population growth, altered land use, deforestation, reduced biodiversity and global trade are leading to an increase in infectious disease incidence and impact [2]. These same factors are also increasing the frequency of disease transmission between humans, domestic animals and wildlife [2, 3] resulting in a dramatic rise in both emerging infectious diseases and zoonoses [4].

Bats, often seen as carriers of many infectious disease agents [5], have also been impacted by this increase in infectious disease incidence, as demonstrated by the recent large scale mortalities caused by white nose syndrome in North America [6]. Other infectious agents have also impacted bat populations. Of particular note is an event involving the mass mortality of thousands of bent-winged bats (*Miniopterus schreibersii*) in France, Spain and Portugal in 2002 [7]. This was thought to have been caused by infection with a novel filovirus, termed Lloviu virus [8], which was only found in dead bats. A betaherpesvirus was also found in the same affected bats. Its significance in the mortality event was unclear, however herpesviruses have been found asymptomatically in a number of different bat species, in addition to bent-winged bats [9–14].

Bent-winged bats are small, cave-roosting, insectivorous bats, weighing between 10 and 19 grams [15]. In south-eastern Australia there are two subspecies of large bent-winged bat (*M*. *orianae*) that form separate maternity colonies [16]. The southern bent-winged bat (*M*. *orianae bassanii*) occurs only in south-western Victoria and south-eastern South Australia (SA). There are two maternity caves, one near Warrnambool (38.3687° S, 142.4982° E) in Victoria and the other near Naracoorte (36.9602° S, 140.7413° E) in SA. In the last 50 years the size of the Naracoorte population of southern bent-winged bats has declined from an estimated 200,000 in the 1950s to 20,000 in 2009 [17]. The Warrnambool population declined from 15,000 to 10,000 over the same time period [17]. The subspecies was listed as critically endangered under the Environment Protection and Biodiversity Conservation Act in 2007. The eastern bent-winged bat (*M*. *orianae oceanensis*) is more common and widespread, being distributed along the east coast of Australia [15]. Although numbers appear to be stable, the subspecies is listed as vulnerable in Victoria due to the use of just a single maternity site [17].

In south-eastern Australia, disease of unknown aetiology has been identified as a possible cause for the dramatic declines in southern bent-winged bat populations [17]. Despite this, there has been only one published disease investigation of this subspecies, at Naracoorte, which examined skin nodules caused by a species of nematode [18]. There has been no systematic survey for viruses to determine if they may be playing a role in the decline.

In addition to herpesviruses and filoviruses, lyssaviruses and adenoviruses are also known to cause disease in bats. Australian bat lyssavirus (ABLV), causes a fatal disease in bats and humans. It is carried by all four species of Australian flying foxes (*Pteropus* spp.), as well as the yellow-bellied sheath-tailed bat (*Saccolaimus flaviventris*) [19]. While previous surveys of bent-winged bats in Australia failed to find any serologically positive animals [19, 20] a serosurvey of common bent-winged bats (*M*. *schreibersii*) in the Philippines found that 36% were positive for neutralizing antibodies against ABLV [21]. Bat-associated adenovirus infections have been mostly asymptomatic [22–25]. The virus was found in the gut of three common pipistrelles (*Pipistrellus pipistrellus*) in Germany with gastrointestinal disease but causation was not established [24].

Bats act as asymptomatic reservoir hosts for a number of important zoonotic viruses that can cause disease or death in humans. This includes the coronavirus that cause Severe Acute Respiratory Syndrome (SARS) [26]. Alpha- and beta-coronavirus RNA and antibodies have been identified in different species of bent-winged bats from northern Australia [27], Thailand [28], Hong Kong [29], China [30] and Bulgaria [31]. Henipaviruses, such as Hendra and Nipah viruses, can also cause severe disease and death in humans [19]. No serological evidence of Hendra virus infection was detected in 62 common bent-winged bats sampled in Queensland, Australia [19], but bent-winged bats that were seropositive for Nipah-like viruses have been identified in China [32]. Filoviruses, including Ebola and Marburg viruses, can cause severe haemorrhagic fevers in humans [33]. More recent publications have identified the presence of a different filovirus (Reston ebolavirus) in three apparently healthy bent-winged bats (*M*. *schreibersii*) in the Philippines [34] and have found serological evidence of infection in two bent-winged bats (*M*. *schreibersii*) in China [35]. The status of Australian bent-winged bats with respect to this viral group is unknown.

The aim of this study was to characterise the virus diversity of the two subspecies to investigate the possibility of viral disease contributing to the decline of the southern bent-winged bat by comparing viruses detected in southern bent-winged bats with those from the more common eastern bent-winged bats. Consequently, we tested populations of southern and eastern bent-winged bats in south-eastern Australia for ABLV, adenoviruses, filoviruses, herpesviruses, coronaviruses and henipaviruses.

## Materials and methods

### Sample collection

Due to concerns that members of the public may enter caves and disturb the critically endangered southern bent-winged bats, this paper uses a generic description of the cave locations, rather than the specific name of each cave.

Sampling was undertaken during summer/early autumn (January-March) and early spring (September), between September 2015 and March 2017. Trapping for southern bent-winged bats occurred at the Naracoorte breeding cave, but, because of the difficult access to the breeding cave near Warrnambool, no trapping occurred there. Instead, those southern bent-winged bats were trapped at nearby non-breeding caves (Allansford (38.3861° S, 142.5931° E), Portland (38.3609° S, 141.6041° E) 1 and 2), with the assumption that these bats were part of the Warrnambool breeding population. Eastern bent-winged bats were trapped at abandoned mines at Christmas Hills (37.6515° S, 145.3173° E) and Eildon (37.2343° S, 145.8976° E) and the only Victorian breeding cave near Lakes Entrance (37.8511° S, 147.9958° E) in eastern Victoria.

Individuals were caught as they flew out of the caves/mines, using harp traps (Austbat, Bairnsdale, Victoria, modified from Tidemann and Woodside, 1978 [36]) set at dusk at the entrances. Traps were monitored continually with the bats either left in the harp trap bag, or transferred in small numbers to cloth bags, prior to sampling. All bats were examined for any external signs of disease, aged as juveniles or adults (based on the presence or absence of a cartilaginous core at the metacarpal-phalangeal joint [37]), sexed, forearm length measured from carpus to elbow, and weighed. In January 2016, only adult bats from Naracoorte were measured and swabbed, but swabs were obtained at the same time opportunistically from juvenile bats which formed part of a different study. The oral cavity was swabbed using sterile, dry, rayon swabs (Copan Flock Technologies, Brescia, Italy). The swab was placed in 500 μL of RNAlater (Life Technologies, Melbourne, Australia) and stored at -20°C until analysis. As part of a larger study, wing swabs were collected for examination for fungi, a maximum of 90 μl blood was taken from the median vein of unanaesthetised bats for haematology, biochemistry and haemoparasite analysis and ectoparasites were collected. Following sampling all bats were released at the point of capture.

During the course of the study 24 dead bats were examined opportunistically (21 from the Naracoorte cave, two from the Warrnambool breeding cave and one from Portland 2 cave), as pathogenic agents may be more likely to be present in dead animals. Bats were necropsied and examined histologically. The causes of death were trauma (11 cases), unknown (nine cases), presumptive bacterial fibrino-suppurative pleuritis (one case), presumptive clostridial septicaemia (one case), parasitism (one case) and inanition (one case). Tissue samples (lung, liver, spleen) were collected for the detection of viruses from seven of these bats (six from the Naracoorte cave and one from the Warrnambool cave). Oral swabs for virus detection were collected from a further 17 of the dead bats (15 from the Naracoorte cave and one each from the Warrnambool cave and Portland 2 cave).

### Extraction of viral DNA and RNA

For viral detection, DNA and RNA were extracted from 200 μL of RNAlater of each swab sample collected from Christmas Hills, Allansford and Naracoorte (January 2016) using VX Universal Liquid Sample DNA Extraction Kits (Qiagen, Melbourne, Australia) and a Corbett X-tractor Gene Robot (Corbett Robotics, Sydney, Australia). Negative extraction controls utilised distilled water (Milli-Q, Merck, USA) only. Positive extraction controls utilised laboratory stocks of cell cultures infected with the avian alphaherpesvirus, infectious laryngotracheitis virus (ILTV) or the avian paramyxovirus, Newcastle disease virus (NDV).

Due to the subsequent unavailability of the X-tractor Gene Robot for extraction, DNA from swab samples collected from Naracoorte (September 2016), Portland 1, Portland 2, Lakes Entrance and Eildon, as well as tissue samples collected from dead bats, was extracted manually using the method described by Steer *et al*. [38]. Manual extraction methods used water (Milli-Q, Merck, USA) as negative extraction control samples, and ILTV infected cell cultures as positive extraction control samples, as described above.

### Detection of Viral DNA and RNA by PCR

For the detection of herpesvirus DNA, extracted DNA was used as a template in a nested pan-herpesviridae PCR, using primers targeting a conserved region of the herpesvirus DNA polymerase gene approximately 200 bp in length [39]. PCR negative controls containing no DNA template, and PCR positive controls containing previously extracted ILTV DNA, were also included. PCR products were visualised on an agarose gel and positive samples underwent DNA purification (QIAquick Gel Extraction Kit, Qiagen, Melbourne, Australia) and Sanger sequencing (Big Dye Terminator version 3.1, Applied Biosystems, Melbourne, Australia).

For detection of adenoviruses and RNA viruses, nucleic acid extracts were submitted to the Victorian Infectious Diseases Reference Laboratory (VIDRL), Melbourne, where they were pooled (five samples per pool) and converted to cDNA using Bioline SensiFast cDNA synthesis kit (Bioline, London, UK) as per the manufacturer’s instructions. The cDNA samples were tested using degenerate consensus PCRs targeting families or genera of the following: *Adenoviridae*, *Coronavirinae*, *Torovirinae*, *Filoviridae*, *Henipaviridae*, lyssavirus and rhabdovirus [40]. The PCR assay positive controls were nucleic acid extracts/cDNA of human adenovirus 5, human coronavirus 229e, Reston ebolavirus, Hendra virus and rabies virus. The assay for *Torovirinae* used an artificial oligonucleotide construct containing the primer binding regions for the respective primers. Successful extraction of RNA was confirmed in positive extraction controls using reverse transcription PCRs targeting conserved motifs in domain III of the paramyxovirus RNA-dependent RNA polymerase gene (applied to cell culture stocks of NDV) or targeting mRNA from the chicken housekeeping gene; glyceraldehyde-3-phosphate dehydrogenase (applied to cell culture stocks of ILTV) [41, 42].

### Sequence and phylogenetic analyses

Herpesvirus sequences were analysed with Geneious 7.1 software (Biomatters Ltd, Auckland, New Zealand). Individual sequences were assigned to groups of identical, or near identical (like) sequences. Near identical sequences were examined for stop codons and multiple peaks. They contained a maximum of four single nucleotide polymorphisms (SNPs) in the approximately 200 bp of the sequenced product (2%). Consensus sequences were generated from groups of like sequences and then compared to published protein sequences in the GenBank database [43] with the highest percentage identity using the BLAST-X algorithm [44]. The resulting predicted amino acid pairwise identities were recorded. A portion of the predicted amino acid sequences of the DNA polymerase region from the detected herpesviruses were aligned with representative members from the three *Herpesviridae* subfamilies from a range of host species using MAFFT v7.308 [45], in conjunction with the BLOSUM62 scoring matrix [46]. The sequences selected for inclusion in this alignment included sequences with a high level of identity to the viruses sequenced in this study, other viruses detected in different bat species, and additional viruses that were selected to ensure that the different *Herpesviridae* subfamilies were represented in the analysis. A phylogenetic tree was generated from the 45 amino acid long alignment using MrBayes v3.2.6 [47] with four heated chains, a chain length of 1,000,000, sampling every 10,000 iterations, and a burn in of 10%. Amino acid sequence is most commonly used when comparing between herpesvirus sub-families as it is typically more informative than relying on overly-divergent nucleotide sequences, which will affect the quality of the alignment and therefore the quality of the tree [48]. Model selection was undertaken using ModelGenerator v0.85 [49], and the Le and Gascuel model [50], with gamma-distributed rate variation across sites and a proportion of invariable sites (LG+I+G), was selected based on the Akaike Information Criterion [51]. A phylogenetic tree was similarly generated using 136 nucleotide sequences, for comparison.

### Statistical analyses

Because of the different properties of betaherpesviruses and gammaherpesviruses [52], these two viral groups were treated separately. However, due to the low number of gammaherpesviruses identified, statistical analyses were confined to betaherpesviruses.

Initial assessment of the data occurred with general descriptive statistics and graphic plots (histograms). Subsequently, a range of potential internal and external predictor variables were screened for association with detection of betaherpesvirus DNA, using univariable logistic regression. These included subspecies, location (grouped as South Australian southern bent-winged bat, Victorian southern bent-winged bat and Victorian eastern bent-winged bat), body mass, sex, age (adult or juvenile) and absence/presence of co-infection with *Polychromophilus* sp. (a haemoprotozoan related to *Plasmodium*), mites, ticks and bat flies (internal factors). Season (spring, summer, autumn) was the only external factor included. Residuals were examined to confirm that model assumptions were met. All factors significant at p < 0.20 were subsequently included in a multivariable logistic regression model, using backward stepping. The final model only included those variables significant at p < 0.05; again, residuals were examined to confirm that model assumptions were met. All statistics were performed using Minitab 18 (Minitab, USA).

## Results

All the initial samples (a total of 213) collected from Christmas Hills, Allansford and Naracoorte (January 2016) were negative for the presence of adenoviruses and RNA viruses (i.e. ABLV, filoviruses, coronaviruses and henipaviruses). Therefore, no further testing for these viruses was undertaken on subsequently collected samples.

In contrast, herpesvirus DNA was detected in 146 (i.e. 31%; n = 467) bats examined. All PCR negative control reactions, and extraction negative control reactions, were negative for the presence of herpesvirus DNA. All positive extraction controls and PCR positive control samples were positive for the presence of the herpesvirus DNA. Herpesvirus DNA prevalence was higher in eastern bent-winged bats (41%; n = 116) than in southern bent-winged bats (27%, n = 351), with the lowest prevalence at Naracoorte (Table 1).

**Table 1:** Prevalence of herpesvirus DNA in oropharyngeal swabs, including proportion of sequence-confirmed positive swabs (confirmed), collected from populations of southern and eastern bent-winged bats from south-eastern Australia. All bats are adults unless otherwise indicated.

Location	Sampling Period	Prevalence % (n)	95% CI	Confirmed
**Southern bent-winged bats**				
Allansford	September 2015	63 (32)	45, 77	16/20
Portland 1	September 2016	38 (45)	25, 52	14/17
Portland 2	February 2017	82 (44)	68, 90	15/36
Naracoorte	January 2016	11 (155) total 8 (63) adults 13 (92) juveniles	7, 17 3, 17 8, 21	17/17
Naracoorte	September 2016	7 (75)	3, 15	3/5
**Eastern bent-winged bats**				
Christmas Hills	September 2015	50 (26)	32, 68	12/13
Eildon	September 2016	18 (39)	7, 30	3/6
Lakes Entrance	March 2017	63 (51) total 81 (31) adults 35 (20) juveniles	49, 75 64, 91 18, 57	9/32

CI–confidence interval.

All the dead bats tested negative for herpesvirus, except for two southern bent-winged bats from Naracoorte, one from a liver sample and another from a lung sample. However, the DNA was of insufficient quality to confirm the positive results by sequencing. All oral swabs collected from dead bats tested negative for herpesvirus DNA.

**Fig 1:**
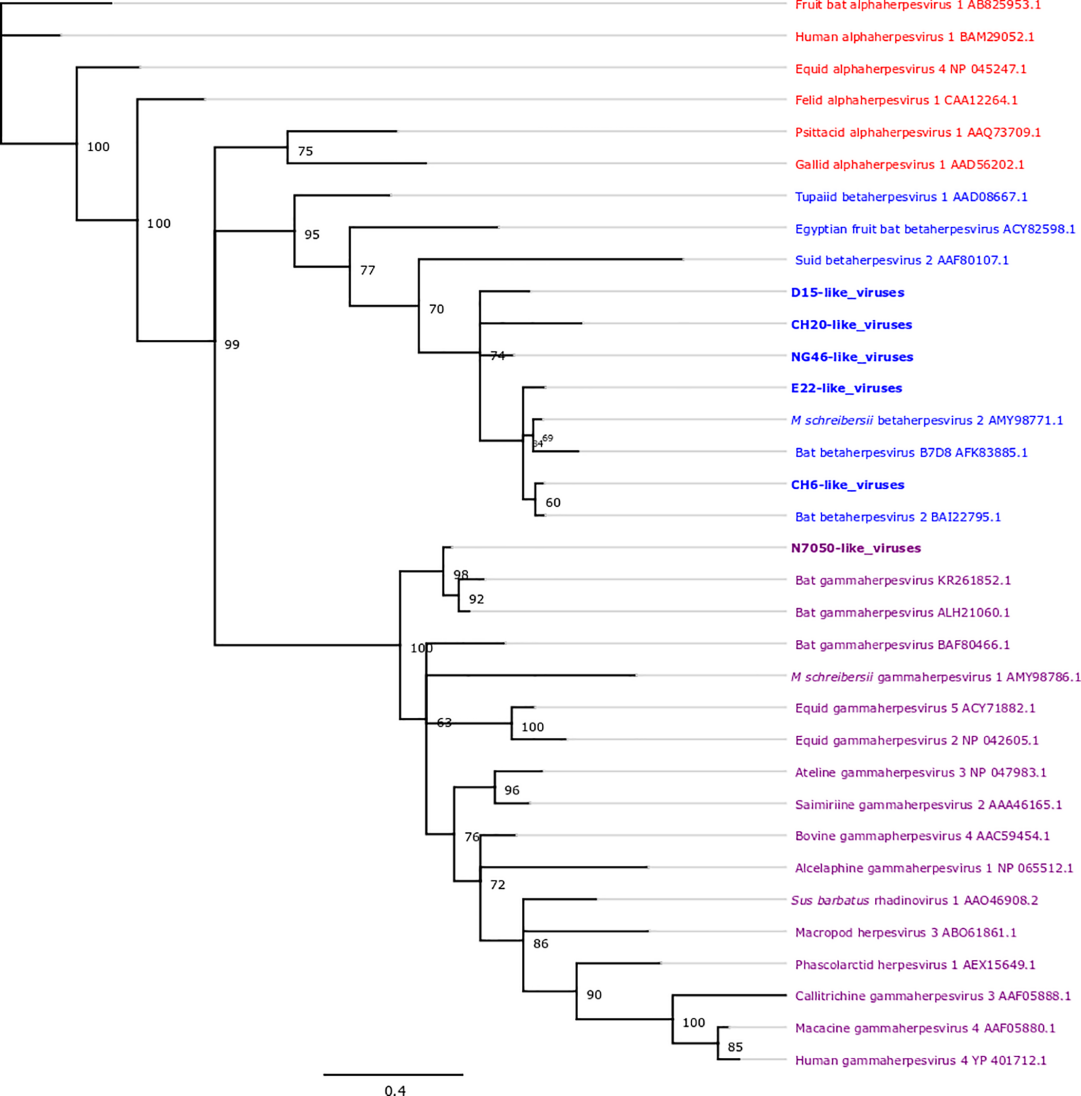
Phylogenetic tree demonstrating the relationship between six bent-winged bat herpesviruses detected in southern and eastern bent-winged bats from south-eastern Australia using PCR, and a selection of viruses from the *Alpha*- (red), *Beta*- (blue) and *Gammaherpesvirinae* (purple) subfamilies. The tree was generated from the 45 amino acid long alignment using MrBayes v3.2.6 [47] with four heated chains, a chain length of 1,000,000, sampling every 10,000 iterations, and a burn in of 10%. GenBank accession numbers are located to the right of each virus.

Herpesvirus DNA suitable for sequencing was obtained from 89/146 of the PCR positive samples. Analysis of the herpesvirus sequence data from these 89 samples revealed six different consensus viral sequences or sequence types (S1 Table), including one gammaherpesvirus (referred to as N7050-like viruses) and five betaherpesviruses (referred to as D15, NG46, CH20, E22 and CH6-like viruses). The viral sequences were compared with bat herpesviruses in GenBank and the maximum percentage predicted amino acid identity to other known bat herpesviruses was found to range from 61% (for CH20-like viruses) to 96% (for CH6-like viruses) (Table 2). This region of the genome is highly conserved in gammaherpesviruses (e.g. 93% amino acid pairwise identity between the closely related equine gammaherpesviruses 5 and 7, GenBank accession numbers AF141886.1 and AKE07652.1, respectively) and betaherpesviruses (e.g. 98% amino acid pairwise identity between the closely related human betaherpesviruses 6A and 6B, GenBank accession numbers APO39176.1 and AB283024.1, respectively), suggesting that the detected viruses may be novel viruses, although further genome sequencing is required to confirm this. A phylogenetic tree demonstrating the relationship of the six virus sequence types with each other and with other known herpesviruses is presented in Fig 1. A similarity matrix of aligned amino acid sequences and a nucleotide phylogenetic tree are available as supplementary information (S2 Table and S1 Fig). This analysis, despite the small fragment of protein sequence available for assessment and low posterior support values for some internal branches, highlighted that the detected viruses clustered within the *Herpesviridae* subfamilies, *Gammaherpesvirinae* and *Betaherpesvirinae*.

**Table 2:** Comparison of bent-winged bat (BWB) herpesvirus sequences, detected by PCR analysis, from southern and eastern bent-winged bats from south-eastern Australia, with the bat herpesvirus in GenBank with the closest identity.

Bent-winged Bat Virus	GenBank Taxonomy ID 668548	Accession Number	Identity	Query/Subject Amino Acids
D15-like	Bat Betaherpesvirus 2	BAI22795.1	75%	41/55
NG46-like	Bat Betaherpesvirus 2	BAI22795.1	82%	47/57
CH 20-like	Bat Betaherpesvirus B7D8	AFK83885.1	61%	31/51
CH 6-like	Bat Betaherpesvirus 2	BAI22795.1	96%	49/51
E 22-like	Bat Betaherpesvirus 2 *Miniopterus schreibersii* Betaherpesvirus 2	BAI22795.1 AMY98771.1	88% 88%	36/41 30/34
N7050-like	Bat Gammaherpesvirus	ALH21055.1	86%	43/50

Pairwise alignments of amino acids were generated using BLAST-X.

After univariable screening, all predictors, except ticks and bat flies, were significant at the p < 0.20 level (Table 3). In the final, multivariable model, location group (p < 0.001), season (p < 0.001), and sex (p = 0.024) were significant predictors of betaherpesvirus shedding (Table 4). Both eastern bent-winged bats and Victorian southern bent-winged bats were significantly more likely to be shedding betaherpesvirus than southern bent-winged bats in South Australia (Odds Ratio, OR = 7.1 and 23.3, respectively), and Victorian southern bent-winged bats were significantly more likely to be shedding than eastern bent-winged bats (OR = 3.3). Bats were more likely to be shedding betaherpesvirus in summer or autumn, and least likely in spring. Males were more likely to be shedding than females (OR = 1.8) (Table 4). Body mass, age and presence of parasites were not significant predictors in the multivariable model.

**Table 3:** Predictor variables screened using univariable logistic regression models for an association with betaherpesvirus infection in southern (SB) and eastern (EB) bent-winged bats from Victoria (Vic) and South Australia (SA), and found to be significant at the p < 0.20 level.

Variable	n	OR	95%CI	p-value
Subspecies (SB)	467	0.467	0.300, 0.725	0.001
Body mass	467	0.767	0.685, 0859	<0.001
Sex (M)	467	1.434	0.959, 2.143	0.079
Age (Juv)	467	0.186	0.094, 0.369	<0.001
Location Group	467			<0.001
Vic EB vs SA SB		11.612	5.942, 22.693	
Vic SB vs Vic EB		2.186	1.302, 3.670	
Vic SB vs SA SB		25.386	13.020, 49.497	
*Polychromophilus* (yes)	264	1.849	1.078, 3.172	0.026
Mites (yes)	376	2.120	1.424, 3.398	<0.001
Season	467			<0.001
Spring vs autumn		0.214	0.112, 0.406	
Summer vs autumn		0.170	0.088, 0.328	
Summer vs spring		0.793	0.506, 1.244	

n = sample size; OR = odds ratio; CI = confidence interval. Reference variable is in brackets.

**Table 4:** Multivariable logistic regression model for betaherpesvirus infection in southern (SB) and eastern (EB) bent-winged bats from Victoria (Vic) and South Australia (SA).

	n	coefficient	SE	OR	95%CI	p-value
Intercept	467	-0.864	0.664			
Location Group						<0.001
Vic EB vs SA SB				7.088	2.719, 18.473	
Vic SB vs SA SB				23.331	10.880, 50.027	
Vic SB vs Vic EB				3.292	1.611, 6.728	
Season						<0.001
Spring vs autumn				0.082	0.031, 0.213	
Summer vs autumn				0.242	0.074, 0.788	
Summer vs spring				2.966	1.450, 6.069	
Male vs female				1.839	1.081, 3.130	0.024

n = sample size; SE = standard error; OR = odds ratio; CI = confidence interval

**Fig 2:**
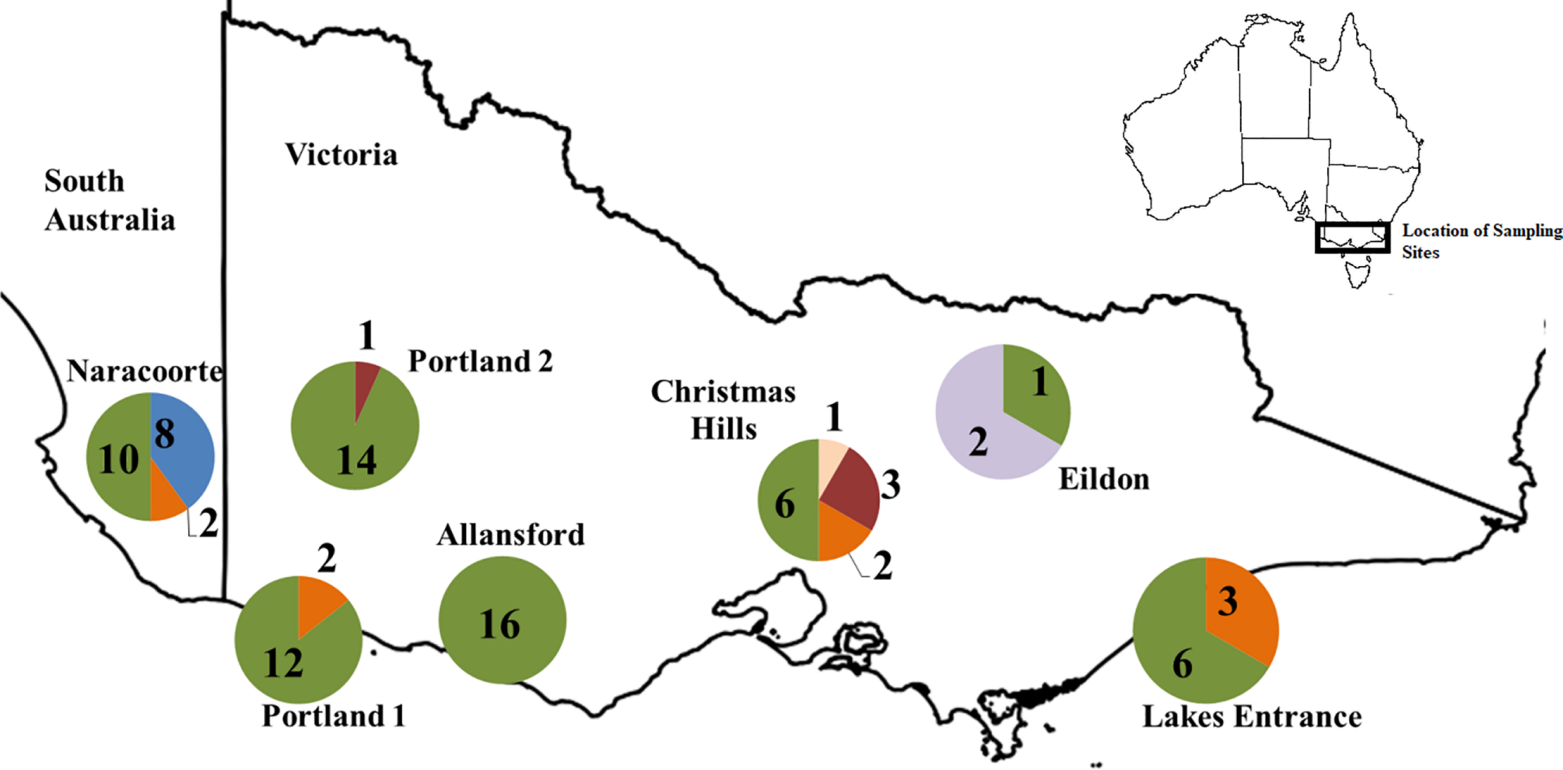
Distribution of herpesviruses detected in each of the seven bent-winged bat locations sampled across Victoria and South Australia (southern bent-winged bat: Naracoorte, Portland 1 & 2, Allansford; and eastern bent-winged bat: Christmas Hills, Eildon and Lakes Entrance). Numbers represent the number of viruses of each type present in the population. D15-like viruses = green. NG46-like viruses = orange. N7050-like viruses = blue. CH20-like viruses = maroon. E22-like viruses = lilac. CH6-like viruses = salmon.

There was geographical and subspecies-specific variation in the distribution of the different viral sequence types (Fig 2). The D15-like betaherpesvirus was found in both bat subspecies and at all locations sampled. In addition, NG46-like and CH20-like betaherpesviruses were found in both subspecies, although not at every location. The N7050-like gammaherpesvirus was only found in southern bent-winged bats, in eight juveniles from Naracoorte, while E22-like and CH6-like betaherpesviruses were only recorded, in small numbers, in eastern bent-winged bats. The eastern bent-winged bats from Christmas Hills contained the largest number of different herpesviruses (four), while the southern bent-winged bats from Allansford only contained one herpesvirus.

## Discussion

This is the most comprehensive viral survey of Australian bent-winged bats. No RNA viruses were found in any of the bats tested. All four RNA virus families tested (ABLV, filoviruses, coronaviruses and henipaviruses) include significant human pathogens. While no serious zoonoses were detected in any of the bats examined in the present study, only a small percentage of the total bat population was sampled, and further research and larger sample sizes are required before concluding that there is no risk to public health.

PCR based assays, as used in this study, generally only detect viruses in animals that have an active infection and are shedding virus at the time of testing [19], while serology will also detect animals that have experienced infections in the past. Due to the small size of bent-winged bats it was not possible to collect sufficient blood to screen for exposure to viruses using the serological tools that were available to us. The small amount of blood that was collected in this study (a maximum of 90 μL per bat) was prioritised for haematological and biochemical testing (as a component of a related study) rather than for virological screening. The use of oral swab samples combined with molecular methods to screen for shedding of viruses has been successfully used in bats for European bat lyssavirus, a virus closely related to ABLV, detecting virus in 15 of 71 samples [53] and consistent with lyssaviruses being shed in the saliva of infected animals [54]. While the present study also used this method to screen for adenoviruses, coronaviruses, filoviruses and henipaviruses, which can all be found in the respiratory and gastrointestinal tracts, testing faeces, urine or blood may have increased the chances of finding these viruses [8, 19, 22, 26, 55].

Six new herpesviruses were detected in the bats examined. This is consistent with recent reports from China and Europe which have revealed a great diversity of herpesviruses in bats, including both betaherpesviruses and gammaherpesviruses [9–14]. Five of the bent-winged bat viruses detected in this study were betaherpesviruses, while one virus, found only in juvenile Naracoorte bats, was a gammaherpesvirus, (N7050-like). This virus had an amino acid sequence identity of 92% to a virus found in Chinese bent-winged bats (Bat Gammaherpesvirus ALH21060.1) [13].

The percentage of bats sampled that were PCR positive for herpesvirus DNA varied widely between the different populations in this study, from 7% to 82%, but this is comparable with other studies of herpesviruses in bats, where the percentage of herpesvirus detection ranged from 3% to 72% [12, 13]. The high prevalence of betaherpesviruses detected in this study may be explained, in part, by the type of samples (oropharyngeal swabs) that were collected. Betaherpesviruses become latent in secretory glands, particularly salivary glands, and would be expected to appear in saliva when excreted [12]. By contrast, a Chinese study that tested bat faecal samples, found a greater proportion of gammaherpesviruses [13]. Faecal samples were not tested in this study but could be considered for future work.

Little is known regarding the extent of movement between the three bent-winged bat populations, although there does appear to be some interchange between them [17]. The different viral diversity profiles present within the bat populations are consistent with restricted, rather than continual, movement of animals between the different locations. Two herpesviruses (CH6-like and E22-like) were detected only in eastern bent-winged bats, whilst another herpesvirus (N7050-like) was detected only in southern bent-winged bats, raising the possibility that these viruses are specific for each of the sub-species of bent-winged bats, although this requires further investigation with larger numbers of animals and samples collected over different times of the year. Three herpesviruses were detected across both bat subspecies, including D15-like herpesvirus, which was found at all seven locations. Herpesviruses have a low rate of nucleotide substitution and have co-evolved with their hosts over many millions of years [56], thus, even in the presence of relatively limited movement of bats between the locations included in the present study, it is expected that the same or similar viruses would occur due to some herpesviruses being present before sub-speciation of the bats, or before bat populations became relatively isolated from each other.

Betaherpesvirus shedding was higher in summer and autumn and lower in spring. Similar patterns have been found in marsupials, humans and cattle [57–59]. When herpes simplex virus was grown in tissue culture, 35°C was found to be the optimum temperature for growth, which ceased below 25°C [60]. As temperatures within the caves occupied by the bats are below 25°C year round [61] and bats go into torpor over winter, lowering their body temperatures to within a few degrees of ambient [62], this would create unfavourable conditions for virus growth and replication. As the bats’ body temperatures and activity levels reach a maximum during the summer months, conditions at this time of year would be more conducive to viral replication and transmission.

Male bats were more likely to be shedding betaherpesviruses than female bats, a pattern which has also been observed in marsupials [57], cats [63] and deer [64]. This may be due to behavioural differences between the sexes affecting rates of transmission, but potential mechanisms for this are unknown.

None of the bats surveyed in this study showed any obvious external signs of ill health or malnutrition. Ideally, ill or recently dead bats would be surveyed as they may contain infectious agents not found in healthy bats, as demonstrated by the European filovirus outbreak [8]. Unfortunately, dead and dying bats are rapidly removed from caves by rats, foxes and other scavengers and rarely found in a state that allows for disease investigation. Based on these results, and the absence of disease or any obvious lesions attributable to herpesvirus infection in any of the bats sampled, it seems likely that the detected herpesviruses are well adapted to their hosts and that bent-winged bats usually carry these herpesviruses with no apparent ill effects.

Latency is an important characteristic of all herpesviruses studied to date and is typically punctuated by intermittent, recurrent viral activation and shedding in response to compromised immunity caused by some form of stress [52]. As southern bent-winged bat numbers have recently declined, it could be assumed that this population is labouring under some form of stress, which may be absent from the eastern bent-winged bat population, which would result in a greater number of herpesvirus positive southern bent-winged bats. While Victorian southern bent-winged bats did have a higher prevalence of infection than eastern bent-winged bats, the opposite was true for the South Australian bats. Similarly, presumably due to an immature immune system [65], more juveniles than adults tend to excrete herpesviruses [52]. This was observed in bats from Naracoorte (southern bent-winged bat), but not those from Lakes Entrance (eastern bent-winged bat).

The PCR used in the present study is able to detect herpesviruses that are being actively shed by the host. However the ability of this PCR to detect latent virus in oropharyngeal swabs cannot be ruled out, as the same nested PCR has been used to detect latent virus genomes in other species [39]. Detection of herpesvirus DNA from both active and latent infection would confound associations between stress/immune status and herpesvirus status because virus detection would not necessarily correlate with virus shedding.

In conclusion, a sample of southern and eastern bent-winged bats was tested by PCR for the presence of six viral families. All bats were negative for adenoviruses, ABLV, filoviruses, coronaviruses and henipaviruses. Six new herpesviruses were identified across all sampled locations. No clinical effects could be attributed to these infections. However, it may be worthwhile conducting further surveys, encompassing a broader range of viruses, because European bent-winged bats (*M*. *schreibersii*) were found to harbour rotaviruses, astroviruses, picornaviruses and bufaviruses [66–69], while other bat species have tested positive for papillomaviruses, polyomaviruses, retroviruses and poxviruses [55, 70].

## Permits

Samples were collected with approval from the Faculty of Veterinary and Agricultural Science Animal Ethics Committee, University of Melbourne, Victoria (ethics approval 1513456.1), Department of Environment, Land, Water and Planning, Victoria (permit number 0007644), Wildlife Ethics Committee, South Australia (permit number 37/2015) and the Department of Environment, Water and Natural Resources, South Australia (permit number Q26488-1).

## Supporting information

S1 Table Nucleotide sequences of herpesviruses identified in this study. (DOCX)

S2 Table Similarity matrix of aligned amino acid sequences. Pairwise alignments of amino acids were generated with MAFFT using the BLOSUM62 matrix. More similar sequences are in green. Less similar sequences are in red.
(XLSX)

S1 Fig Phylogenetic tree demonstrating the relationship between six bent-winged bat herpesviruses detected in southern and eastern bent-winged bats from south-eastern Australia using PCR, and a selection of viruses from the *Alpha*- (red), *Beta*- (blue) and *Gammaherpesvirinae* (purple) subfamilies. The tree was generated from 136 nucleotide sequences aligned with MAFFT [45] and substitution model selection (using AIC) undertaken with ModelGenerator [49]. The tree was built using MrBayes [47] with four heated chains and a burn in of 10%, subsampled every 10,000 and using the general time reversible model with gamma-distributed rate variation across sites and a proportion of invariable sites (GTR+I+G) [71]. GenBank accession numbers are located to the right of each virus.
(TIF)
