# Supplementary material for: Virus survey in populations of two subspecies of bent-winged bats (Miniopterus orianae bassanii and oceanensis) in south-eastern Australia reveals a high prevalence of diverse herpesviruses
Source: PLoS One. 2018 May 24;13(5):e0197625. doi: 10.1371/journal.pone.0197625 (PMC5967723; doi:10.1371/journal.pone.0197625)
Supplement: S1 Table — (DOCX) [file pone.0197625.s001.docx]

| **Virus** | **Nucleotide Sequence** |
| --- | --- |
| N7050-like | AGAGAAGAGTTCACTCTTCAGGGGAGAACATGCTGGAGAAGACAAAAAATTACATTGAGGCAGTCACTCCAGAAAGGCTTCATGAAATTGTCCCTGTCCCCTTCAATCACGACCCAGATGCTCACTTCAAAGTCATATACGGAGACACGGACTCTGTGA |
| D15-like | GCCTGCCTGGCGTCGCGGCGTCGATAACGCGGATAGGTCGGGACATGCTGTCGAGGACGATGGAGTACATCCACTCGCACCTGTCCGATCGTTCCGGTCTGTGCGCGTTTTTTAACGACGCCGACTACCTGCCCGACTCCGAACTGTCCGTTCGCGTCATCTACGGCGACACGGACTCTGTGA |
| NG46-like | CCGATTCGGCGATGATACTCTGTCTAGTGCATCGCGGCGTCGATAACCAGGATCGGTCGCGATATGCTCTCGAGCACGGCTAACTATATTCATTCTCACCTGTCCGATCGTTCCGGACTGTCGGCGTTCTTCGCCGAAACCGACTACGTATCCGACGAAGGCGTGTCGGTGCGTGTCATCTACGGCGACACGGACTCTGTGGA |
| CH20-like | ACGCGCATAGGTCGTGGCATGCTGTCTTCCACGGCAGAATATATCCACAGGAGCCTATCGGACCGTGAAATGCTATCAAAATTTATAAACGAAGAAGATTTTATTGCAGGCGAAGAGGTTTCTGTAAAAGTAATCTACGGCGACACGGACTCTGTGA |
| E22-like | GTGATCGCGCGTCGATTAGCGTGATAGGGCGGGACATGCTCTACGCGAACGGCGAGTTACATCCATTCTAACCTCTGCGATCGCGCCGGGCTAGGGGCGTTTTTTACCGAGGCGGACTACATTCCGGACGAGACCGTGTCCGTGCGGGTCATCTACGGCGACACGGACTCTGTGA |
| CH6-like | ACTCTGTGACACCAGGATAGGGCGAGATATGTTGTCGAAGACGGCGAGTTACATCCATTCTAACCTCTCTGACCGCGCCGGGCTCGATGCGTTTTTCACCGAGGCCGACTATGTTCCTGACGAGAGCGTGTCGGTGCGGGTCATCTACGGCGACACGGACTCTGTGA |
