# Supplementary figures and images for: Virus survey in populations of two subspecies of bent-winged bats (Miniopterus orianae bassanii and oceanensis) in south-eastern Australia reveals a high prevalence of diverse herpesviruses
Source: PLoS One. 2018 May 24;13(5):e0197625. doi: 10.1371/journal.pone.0197625 (PMC5967723; doi:10.1371/journal.pone.0197625)

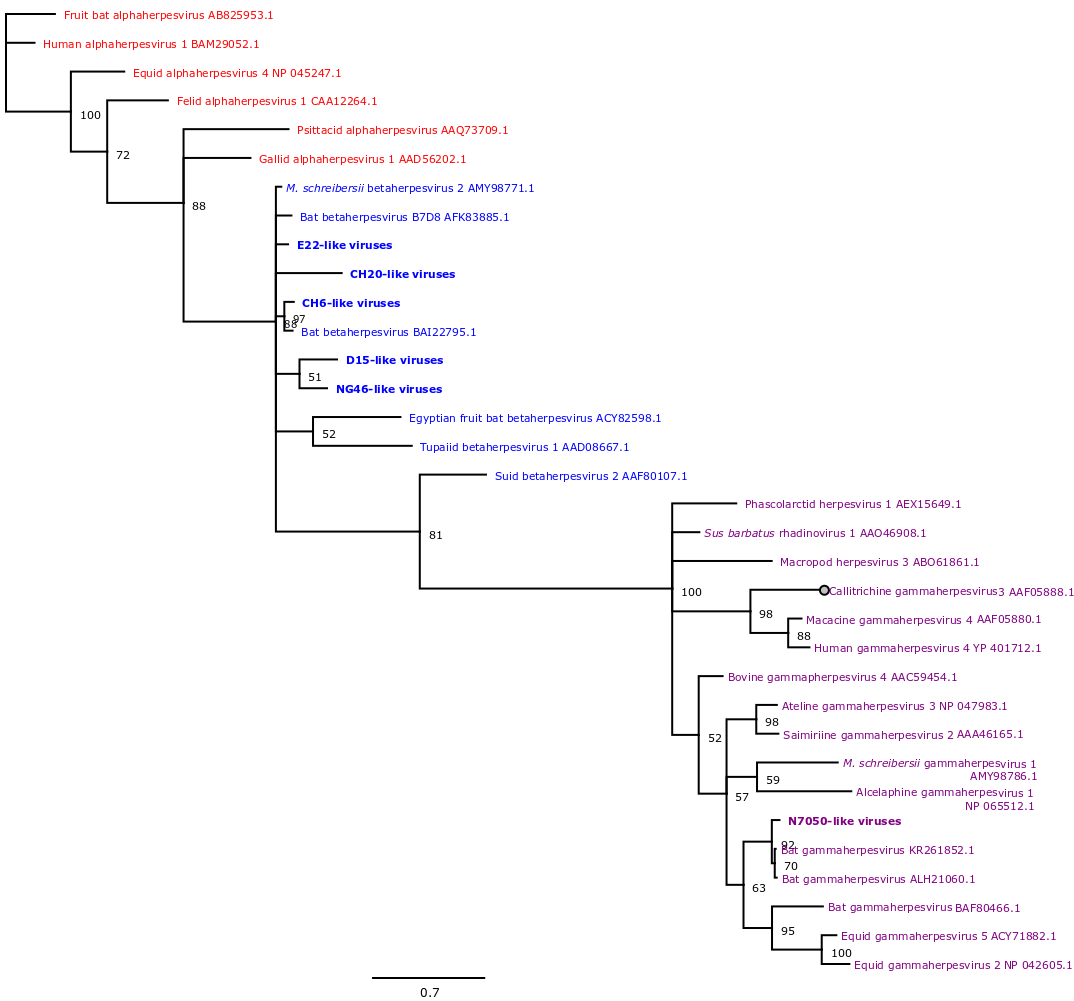

Supplement: S1 Fig — The tree was generated from 136 nucleotide sequences aligned with MAFFT [45] and substitution model selection (using AIC) undertaken with ModelGenerator [49]. The tree was built using MrBayes [47] with four heated chains and a burn in of 10%, subsampled every 10,000 and using the general time reversible model with gamma-distributed rate variation across sites and a proportion of invariable sites (GTR+I+G) [71]. GenBank accession numbers are located to the right of each virus. (TIF) [file pone.0197625.s003.tif]
